# Modelling residual disease volume and re-growth rates from response durations to aid in cancer trial design, interpretation and analysis, with a particular focus on breast cancer

**DOI:** 10.1186/1745-6215-14-S1-O120

**Published:** 2013-11-29

**Authors:** Walter Gregory, Helen Marshall, David Cameron, Christopher Twelves, Richard Bell, Robert Coleman

**Affiliations:** 1 University of Leeds, Leeds, West Yorkshire, UK; 2 University of Sheffield, Sheffield, South Yorkshire, UK; 3 University of Edinburgh, Edinburgh, Scotland, UK; 4 Andrew Love Cancer Centre, Geelong, Victoria, Australia

## Background

There is what might be termed an analysis gap between scientific developments and understandings at the cellular and genetic levels which lead to new cancer treatments and conventional statistical trial designs and analyses that focus on demonstrating superiority or non-inferiority.

## Aim

To develop and apply multivariate mathematical/statistical models that infer cell-kill and tumour re-growth dynamics from durations of remission/response to cancer treatment.

## Methods

A multivariate mathematical model was developed based on a series of assumptions about the volume and distribution of residual disease remaining after treatment at the population level, and its subsequent regrowth. This model enables the results of randomised clinical trials to be evaluated in terms of effects on these biologically relevant parameters.

## Results

Examples will be given in a number of diseases but focusing on breast cancer and the results from the AZURE trial on the use of zoledronic acid, as well as from CALGBB trial 9741 which evaluated a dose-dense treatment strategy, as proposed by Norton and Simon. Results will be presented, including quantification of the cell-killing effect of zoledronic acid from the AZURE trial, which demonstrate how the additional understandings gained from such a modelling approach can aid in trial design, interpretation of results, monitoring and analysis.

## Conclusions

This modelling approach leads to new insights on the mechanism of action of cancer treatments which stimulates the development of new innovative approaches to treatment and more appropriate targeting of treatments to particular subgroups of patients.

